# Gene selection for classification of microarray data based on the Bayes error

**DOI:** 10.1186/1471-2105-8-370

**Published:** 2007-10-03

**Authors:** Ji-Gang Zhang, Hong-Wen Deng

**Affiliations:** 1 Laboratory of Molecular and Statistical Genetics, College of Life Sciences, Hunan Normal University, Changsha, Hunan 410081, P. R. China; 2 The Key Laboratory of Biomedical Information Engineering of Ministry of Education and Institute of Molecular Genetics, School of Life Science and Technology, Xi'an Jiaotong University, Xi'an 710049, P. R. China; 3 Departments of Orthopedic Surgery and Basic Medical Science, School of Medicine, University of Missouri-Kansas City, 2411 Holmes Street, Kansas City, MO 64108, USA

## Abstract

**Background:**

With DNA microarray data, selecting a compact subset of discriminative genes from thousands of genes is a critical step for accurate classification of phenotypes for, e.g., disease diagnosis. Several widely used gene selection methods often select top-ranked genes according to their individual discriminative power in classifying samples into distinct categories, without considering correlations among genes. A limitation of these gene selection methods is that they may result in gene sets with some redundancy and yield an unnecessary large number of candidate genes for classification analyses. Some latest studies show that incorporating gene to gene correlations into gene selection can remove redundant genes and improve classification accuracy.

**Results:**

In this study, we propose a new method, Based Bayes error Filter (BBF), to select relevant genes and remove redundant genes in classification analyses of microarray data. The effectiveness and accuracy of this method is demonstrated through analyses of five publicly available microarray datasets. The results show that our gene selection method is capable of achieving better accuracies than previous studies, while being able to effectively select relevant genes, remove redundant genes and obtain efficient and small gene sets for sample classification purposes.

**Conclusion:**

The proposed method can effectively identify a compact set of genes with high classification accuracy. This study also indicates that application of the Bayes error is a feasible and effective wayfor removing redundant genes in gene selection.

## Background

One of the major applications of DNA microarray technology is to perform sample classification analyses between different disease phenotypes, for diagnostic and prognostic purposes [1-3]. The classification analyses involve a wide range of algorithms such as differential gene expression analyses, clustering analyses and supervised machine learning [4-6], etc. In classification analyses of microarray data, gene selection is one of the critical aspects [5,7-12]. Efficient gene selection can drastically ease computational burden of the subsequent classification task, and can yield a much smaller and more compact gene set without the loss of classification accuracy [13-17]. In addition, a smaller number of selected genes can be more conveniently and economically used for diagnostic purposes in clinical settings [18].

In the presence of thousands of genes in microarray experiments, it is common that a large number of genes are not informative for classification because they are either irrelevant or redundant [19]. Based on a review of the definitions of relevance [20,21], the genes can be classified into three disjoint categories, namely, strongly relevant, weakly relevant, and irrelevant genes [21]. Strong relevance indicates that the gene is always necessary for an optimal subset and cannot be removed without affecting the classification accuracy. Weak relevance indicates that the gene is not always necessary but may become necessary for an optimal subset at certain conditions. Irrelevance indicates that the gene is not necessary at all for classification accuracy. Hence, an optimal gene subset should include all strongly relevant genes, none of irrelevant genes, and a subset of weakly relevant genes. Based on the relevance definitions of genes, in classification analyses, a redundant gene is the one (which may be useful for classification analyses in isolation) does not provide much additional information if another informative gene is already present in the chosen gene subset.

Various approaches have been developed for gene selection to extract relevant genes from thousands of genes in microarray experiments, such as clustering methods [22] and pair-wise correlation analyses [2,6,23]. While multiple methods are available, it is well accepted in the field that a good gene selection method should be able to: 1) simplify the classifier by retaining only the relevant genes [23,24]; 2) improve or not significantly reduce the accuracy of the classifier; and 3) reduce the dimensionality of the dataset [9,25-27].

Traditionally, the methods for gene selection are broadly divided into three categories: filter, wrapper and embedded methods [21]. A filter method relies on general characteristics of the training data to select genes without involving any classifier for evaluation [10,28]. Many filter methods are usually mentioned as individual gene-ranking methods [1,10,26,29,30]. They evaluate a gene based on its discriminative power for the target classes without considering its correlations with other genes. Although such gene ranking criteria are simple to use, they ignore correlation among genes, which may result in inclusion of redundant genes in selected gene set used for classification [31]. Redundant genes will increase the dimensionality of the selected gene set, and in turn affect the classification performance, especially on small samples [26]. In order to minimize redundant genes, correlation analyses have been incorporated in gene selection to remove redundant genes and improve classification accuracy [23,24,26,32]. The wrapper methods utilize the classifiers as evaluation functions and search for the optimal gene set for classification [14]. But the wrapper methods may suffer from excessive computational complexity. In contrast to the filter and wrapper approaches, the embedded methods perform the selection of genes during the training procedure and are specific to the particular learning algorithms [14,21,24,33]. For the wrapper and embedded methods, the search schemes are always involved to identify the optimal gene set for the sample classification. Searching the whole gene subset space may discover the optimal gene subset with respect to an evaluation criterion. However, an exhaustive search is usually computationally prohibitive. Thereby some partial search schemes are proposed, such as sequential forward selection, sequential floating forward selection, sequential backward elimination, sequential floating backward elimination and random search [34]. These partial search schemes are practically more feasible but provide no guarantee for identifying the optimal gene set [33].

In the earlier literatures, some excellent studies have highlighted the advantages of controlling classification error in yielding an optimal gene set, such as the study in the reference [35]. In this article we develop a novel gene selection method based on the Bayes error. Although the Bayes error has been used for feature selection in classification analyses, its use for gene selection in microarray data is very rare. It is well known that the Bayes error can provide the lowest achievable error rate bound for a given classification problem [36]. Theoretically, the Bayes error is the best criterion to evaluate effectiveness of gene set for classification [37], and the Bayes error depends only on the gene space, not the classifier itself [38]. From this point of view, by controlling the Bayes error it is feasible to find the optimal or sub-optimal gene set for a given classification problem without designing the classifiers. However, it is usually difficult to estimate directly the Bayes error rate analytically. An alternative is to estimate an upper bound of the Bayes error, which could be obtained by an error estimation equation based on the Bhattacharyya distance [37,39]. With this method, we can indirectly use the Bayes error for gene selection by controlling the upper bound of the Bayes error. This strategy is more promising than those requiring gene selection and classifier design simultaneously, as in the wrapper methods. Considering the promising aspects of the Bayes error, we propose in this study an approach, BBF (Based Bayes error Filter), for gene selection. Our selection algorithm is implemented in two steps: 1) first the relevant candidate genes are selected by a criterion function; and 2) the criterion controlling the upper bound of the Bayes error is applied to the relevant candidate genes in order to remove the redundant genes.

## Application

To evaluate the performance of our proposed method in practice, we analyzed five publicly available microarray datasets: 1) Colon cancer dataset; 2) DLBCL dataset; 3) Leukemia dataset; 4) Prostate dataset; 5) Lympho ma dataset (see Table 1).

**Table 1:** Summary for five datasets used in our experiments

Dataset	Source	No. of genes	No. of samples	Classes
Colon	Alon *et al. *(1999)	2000	62	Normal/Tumor
DLBCL	Shipp *et al. *(2002)	6285	77	DLBCL/FL
Leukemia	Golub *et al. *(1999)	3051	38	ALL/AML
Prostate	Singh *et al. *(2002)	6033	102	Normal/Tumor
Lymphoma	Alizadeh *et al. *(2000)	4026	47	Germinal/Activated

### Colon cancer dataset

This dataset consists of expression levels of 62 samples of which 40 samples are colon cancer samples and the remaining are normal samples [13,40]. Although originally expression levels for 6,000 genes are measured, 4,000 genes out of all the 6,000 genes were removed considering the reliability of measured values in the measured expression levels. The measured expression values of 2,000 genes are publicly available at [41].

### DLBCL dataset

This dataset contains 77 samples in two classes, diffuse large B-cell lymphomas (DLBCL) and follicular lymphoma (FL), which have 58 and 19 samples, respectively [42]. The original dataset contains 7,129 genes. After the quality control, the dataset contains 77 samples and 6,285 genes. The measured expression values of genes are available at [30].

### Leukemia dataset

This dataset, provided by Golub *et al. *[43], contains the expression levels of 7,129 genes for 27 patients of acute lymphoblastic leukemia (ALL) and 11 patients of acute myeloid leukemia (AML). After data preprocessing, 3,051 genes remain. The source of the 3,051 gene expression measurements is publicly available at [44].

### Prostate dataset

This data set provides the expression levels of 12,600 genes for 50 normal tissues and 52 prostate cancer tissues [45]. The experiments were run on Affymetrix human 95Av2 arrays. The data preprocessing step leaves us with 6,033 genes. The data source is available at [46].

### Lymphoma dataset

This dataset presented by Alizadeh *et al. *[47] comprises the expression levels of 4,026 genes. It contains 47 samples and two classes: germinal center B cell-like DLCL (diffuse large cell lymphoma) and active B cell-like DLCL. Among the 47 samples, 24 samples are germinal center B-like DLCL and 23 samples are active B cell-like DLCL. The dataset is available at [48].

## Results

In the gene preselection step, we select the genes with FWER ≤ 0.05 (Family-Wise-Error rate). In our experiments, KNN and SVM classifiers are employed to demonstrate the proposed method and its classification performance. We choose the Euclidean distance in our KNN classifier with K = 5 and predict the class label by a majority vote. For the SVM classifier, we choose a linear kernel for decision plane computation.

We assess the performance of our method using the "Leave-One-Out Cross Validation" (LOOCV). LOOCV provides realistic assessment of classifiers which generalize well to new data [27]. The LOOCV method proceeds as follows: hold out one sample for testing while the remaining samples are used to make the gene selection and train the classifier. Note that to avoid selection bias [49], gene selection is performed using the training set. The genes are selected by our method using the training samples and then are used to classify the testing sample. The overall test error rate is calculated based on the incorrectness of the classification of each testing sample. Table 2 summarizes classification errors of five datasets with KNN and SVM classifiers by our method.

**Table 2:** The LOOCV errors for two-class datasets using KNN and SVM

Dataset	KNN(K = 5)		SVM(Linear)	
	Number of genes	Lowest Error (%)	Number of genes	Lowest Error (%)
Colon	12	9.68	20	12.90
DLBCL	6	7.79	5	9.09
Leukemia	3	0.00	2	0.00
Prostate	11	5.88	13	3.92
Lymphoma	8	2.13	3	0.00

For the Colon dataset, as shown in Table 2, using the BBF method, 6 out of 62 samples are incorrectly classified by KNN and 8 by SVM, resulting in an overall error rate of 9.68% and 12.90%, respectively. According to the results of Ben-Dor *et al. *[13], without gene selection the classification error was 19.35% for KNN, and 22.58% for SVM, respectively. This is a significant improvement compared to the accuracy obtained by all available genes. This implies that there are irrelevant or redundant genes which deteriorate the performance of the classifiers, and the appropriate gene selection could effectively improve classification accuracy. The colon dataset has been used by many studies. For example, Liu *et al. *[23], used "normalized mutual information" with greedy selection and simulated annealing algorithm for gene selection. They reported that using KNN classifier the classification error is 9.68% with 29 selected genes for greedy selection and 12.90% with 26 selected genes for simulated annealing algorithm. Ding and Peng [26] proposed a "Minimum Redundancy – Maximum Relevancy" (MRMR) method. Their best result by SVM was 8.06% with 20 genes, which means 5 out of 62 samples are incorrectly classified. Compared with our results, Liu's method [23] used more genes for similar classification errors. Using the SVM classifier, Ding and Peng [26] also selected 20 genes for best classification accuracy and the accuracy is slightly higher than ours. Some studies demonstrate that accurate diagnoses could be achieved using the expression levels of 15–20 genes from colon dataset [50]. To sum up the above results, the necessary number of genes could be less than 20 for the Colon dataset.

For the DLBCL dataset, in the original article, Shipp *et al*. [42] picked 30 genes by using their own weighted combination of informative genes. They correctly classified 71 out of 77 patients for a diagnostic error of 7.79%. Using our method 6 out of 77 samples are incorrectly classified by KNN and 7 by SVM, resulting in an overall error rate of 7.79% and 9.09%, respectively. However, only 5–6 genes are involved in classification and obtain similar classification error. Yang, *et al *[30] proposed GS1 and GS2 methods based on the ratio of inter-class and intra-class variation as a criterion function for gene selection. Using KNN they obtained classification error rate of 7.79% with 85 genes by GS1 and 6.49% with 70 genes by GS2; using SVM they achieved classification error rate of 3.90% for GS1 with 81 genes and GS2 with 55 genes. In contrast, we note that our results with KNN method are almost as good as theirs, yet only 6 genes are involved in the classification procedure and our classification error with SVM method is slightly higher than their results, but we use only 5 genes to reach similar level of performance.

For the Leukemia dataset, using the BBF method, all samples are correctly classified by KNN and SVM, with 3 and 2 genes for the two classifiers, respectively. Compared with other gene selection methods, our method appears to yield higher classification accuracy. For example, Dettling and Buhlmann [51] adopted four classifiers (LogitBoost, AdaBoost, KNN and Classification tree) to classify Leukemia dataset, and classification error was calculated based on LOOCV method. Their best classification error result was 1.39% with 25 genes by KNN. Some studies also come up with similar results to ours. Weston *et, al *[52] reported 0% classification error for a linear SVM using 20 genes by LOOCV method, but they used more genes.

For the Prostate dataset, in our results, 6 out of 102 samples are incorrectly classified by KNN and 4 by SVM, resulting in an overall error rate of 5.88% and 3.92%, respectively. Dettling and Buhlmann [22] proposed an algorithm for selecting supervised clusters of genes to find the gene groups for classification. They used KNN and aggregated trees methods, achieving the best results of classification error being 4.9% with 3 gene clusters by KNN. Our results are comparable to theirs, but used fewer genes. Gentile [53] used on a incremental large margin algorithm for gene selection and yielded 6.5% classification error estimated with 100 genes by LOOCV method. In contrast, we only used 11–13 genes and achieved better accuracy.

For the Lymphoma dataset, using our method, the classification error rates of KNN and SVM are 2.13% (this means only one sample is incorrectly classified) and 0%, respectively. Wang *et, al. *[54] also analyzed this dataset using several different gene selection methods and classifiers by the LOOCV method. In their study, they combined the locally linear embedding method and SVM classifier and yielded the classification error of 8.5% with 50 selected genes. When combining Signal-to-Noise method with KNN classifier, the classification error was 23.4%. The best error rate result they reported was 4.35% with 20 genes which is obtained by adopting the Information Gain method and a Neuro-Fuzzy Ensemble model. Diaz-Uriarte and Alvarez de Andres [55] reported the similar results to ours with the random forest method for gene selection, though they used a different estimation method for classification error rate. But they used more genes than our method. The results of our BBF method are comparable or outperform the above other results.

## Discussion

As an important statistical index for classification analyses [56,57], the Bayes error has rarely been used in classification analyses of microarray data. In this study, we propose a novel gene selection approach for microarray classification analyses. We introduce the Bayes error into the gene selection procedure, which turned out to be beneficial for classification analyses. The experimental results show that our proposed method can 1) reduce the dimension of microarray data by selecting relevant genes and excluding the redundant genes; and 2) improve or be comparable to the classification accuracy compared with other earlier studies.

In classification analyses, the classification error is an important criterion for selection of an optimal gene set. Some gene selection methods have been proposed to achieve the minimum classification error. Among these methods, a typical one is the method proposed by Peng *et al. *[35] which incorporates the classification error estimation with the gene selection method to determine an optimal gene set. The method first selects the genes with the highest relevance to the target classes, and minimizes the redundancy among the selected genes, and then determines an optimal gene set which has the minimum classification error estimated by cross-validation methods. In addition, the researchers presented the theoretical analysis [35]and comprehensive experimental studies [26] to prove that the criterion of "Maximum Relevance and minimum Redundancy" can benefit selection of optimal features for classification. Similar to the method of Peng *et al. *[35], our method uses the Bhattacharyya distance to select the genes with the highest joint relevance to the target classes, while minimizing the redundancy among the selected genes. Meanwhile, we can indirectly control the classification error due to the relationship between the Bhattacharyya distance and the Bayes error, and thus we can effectively avoid the computation of cross-validation error.

From the Bayesian decision theory, it is known that 1) the probability of error of any classifier is lower bounded by the Bayes error, 2) the Bayes error only depends on the gene space, not the classifier itself, and 3) there is always at least one classifier that achieves this lower bound [36,57,58]. Hence, from a theoretical point of view, it is possible to find out an optimal gene set for a given classification problem, rendering the minimum classification error. When selecting a set of relevant genes G with a minimum Bayes error in all gene space, according to the theories of the Bayes error, it can be guaranteed that at least one classifier may achieve this classification error. However, it should be noted that this optimal relevant gene set is classifier-specific. As pointed out [21,23], there are no relevancy definitions independent of the classifiers. That means not all classifiers can achieve the minimum Bayes error with the gene set G. This is because different classifiers have different biases and a gene which may be favorable for one classifier but may not for another. This phenomenon is also observed in our results. For example, in the colon dataset, for SVM classifier the best classification error was 12.90% with 20 genes, while for KNN classifier we could achieve the best classification error with 12 genes. When more genes are involved, the error rate for KNN classifier will increase. Since no single subset is optimal for all classifiers, it would be sensible to adopt a strategy to incorporate a classifier into gene selection for classification like a wrapper method. As the two-stage algorithm proposed in the early study [35], the first stage is to select relevant genes and eliminate redundant genes; the second stage is to search a more compact gene set for a specific classifier. This algorithm may not only yield an optimal or sub-optimal gene set for a specific classifier and increase the classification accuracy, but also decrease computation complexity when compared to a wrapper method. Our method can be extended to adopt this algorithm.

In classification analyses, genes obtained from observations may not be all informative for target classes. It is necessary to pick out the relevant candidate genes even though some of them are redundant. For our gene selection system, a gene preselection step is used to select the relevant candidate genes based on their individual relevance to the target classes. But the gene preselection step alone cannot yield the optimal gene set for a classification problem because it cannot eliminate the redundant genes due to the correlations between genes [4,5,36]. Efforts have been made to minimize the redundancy by measuring pair-wise gene correlations within the selected gene set [26,32,35,59], performing clustering analyses [23,26] and Markov blanket filtering [60]. Our proposed BBF method identifies the redundant genes by using Bhattacharyya distance measure to minimize the Bayes error. It is clear from Equation 2 that if a gene is highly correlated with another gene, combination of these two genes may not contribute more to the Bhattacharyya distance measure between two classes than any one of them. For an extreme example, i.e., the correlation of two genes is 1, it is impossible to calculate inverse matrix of covariance matrix for Bhattacharyya distance measure between two classes, and thus the redundant gene can be eliminated.

In addition, upper bound of the Bayes error (denoted by ε*_B_) is a critical parameter in this gene selection scheme. In the BBF method, we select genes according to their contribution to Bhattacharyya distance, *d*_*B*_. It has been proved that ε*_B _monotonically increases as *d*_*B *_increases, but at a decelerating manner since the rate of increase of ε*_B _decreases with the increasing of *d*_*B *_[36]. When *d*_*B *_increases to a certain level, e.g., 4.0, increasing *d*_*B *_may not efficiently improve classification accuracy. Under this condition, with more genes selected, their contributions to classification accuracy turn out to be increasingly negligible. In the case of high ε*_B_, it may lead to a loss of some relevant genes; in the case of smaller ε*_B_, it may involve some genes of negligible effects for classification. With this in mind, we set a criterion, ε*_B _being 1.0E-4, for picking relevant genes.

## Conclusion

In summary, the BBF method can effectively perform gene selection with reasonably low classification error rates and a small number of selected genes. Our method may not only obtain a small subset of informative genes for classification analyses, but also provide a balance between selected gene set size and classification accuracy. This is confirmed by testing our method on the 5 real datasets.

## Methods

In microarray classification analyses, the main objective of gene selection is to search for the genes which keep the maximum amount of information about the class and minimize the classification error. According to the Bayes theorem, the Bayes error can provide the lowest achievable error rate bound for a given classification problem (see details in [61,62]). The problem of gene selection is equivalent to determining the subset of genes which can minimize the Bayes error.

Let us consider the situation where a given gene expression measurement vector ***x ***needs to be classified into one of *L *classes. *P(c*_*i*_) denotes the a priori class probability of class *i*, 1 ≤ *i *≤ *L*, and *p*(*x*|*c*_*i*_) denotes the class likelihood, i.e., the conditional probability density of ***x ***given that it belongs to class *i*. The probability of ***x ***belonging to a specific class *i*, i.e., the posteriori probability *p*(*c*_*i*_|*x*), is given by the Bayes theorem:

p(ci|x)=p(x|ci)P(ci)p(x)
 MathType@MTEF@5@5@+=feaafiart1ev1aaatCvAUfKttLearuWrP9MDH5MBPbIqV92AaeXatLxBI9gBaebbnrfifHhDYfgasaacH8akY=wiFfYdH8Gipec8Eeeu0xXdbba9frFj0=OqFfea0dXdd9vqai=hGuQ8kuc9pgc9s8qqaq=dirpe0xb9q8qiLsFr0=vr0=vr0dc8meaabaqaciaacaGaaeqabaqabeGadaaakeaacqWGWbaCdaqadaqaaiabdogaJnaaBaaaleaacqWGPbqAaeqaaOGaeiiFaWNaemiEaGhacaGLOaGaayzkaaGaeyypa0ZaaSaaaeaacqWGWbaCdaqadaqaaiabdIha4jabcYha8jabdogaJnaaBaaaleaacqWGPbqAaeqaaaGccaGLOaGaayzkaaGaemiuaa1aaeWaaeaacqWGJbWydaWgaaWcbaGaemyAaKgabeaaaOGaayjkaiaawMcaaaqaaiabdchaWnaabmaabaGaemiEaGhacaGLOaGaayzkaaaaaaaa@4955@

where *p*(*x*) is the probability density function of *x *and is given by:

p(x)=∑i=1Lp(x|ci)P(ci)
 MathType@MTEF@5@5@+=feaafiart1ev1aaatCvAUfKttLearuWrP9MDH5MBPbIqV92AaeXatLxBI9gBaebbnrfifHhDYfgasaacH8akY=wiFfYdH8Gipec8Eeeu0xXdbba9frFj0=OqFfea0dXdd9vqai=hGuQ8kuc9pgc9s8qqaq=dirpe0xb9q8qiLsFr0=vr0=vr0dc8meaabaqaciaacaGaaeqabaqabeGadaaakeaacqWGWbaCdaqadaqaaiabdIha4bGaayjkaiaawMcaaiabg2da9maaqahabaGaemiCaa3aaeWaaeaacqWG4baEcqGG8baFcqWGJbWydaWgaaWcbaGaemyAaKgabeaaaOGaayjkaiaawMcaaiabdcfaqnaabmaabaGaem4yam2aaSbaaSqaaiabdMgaPbqabaaakiaawIcacaGLPaaaaSqaaiabdMgaPjabg2da9iabigdaXaqaaiabdYeambqdcqGHris5aaaa@472E@

When assigning a vector ***x ***to the class with the highest posterior probability, the error associated with this classification is called the Bayes error, which can be expressed as:

Ebayes=1−∑i=1L∫CiP(ci)p(x|ci)dx
 MathType@MTEF@5@5@+=feaafiart1ev1aaatCvAUfKttLearuWrP9MDH5MBPbIqV92AaeXatLxBI9gBaebbnrfifHhDYfgasaacH8akY=wiFfYdH8Gipec8Eeeu0xXdbba9frFj0=OqFfea0dXdd9vqai=hGuQ8kuc9pgc9s8qqaq=dirpe0xb9q8qiLsFr0=vr0=vr0dc8meaabaqaciaacaGaaeqabaqabeGadaaakeaacqWGfbqrdaWgaaWcbaGaemOyaiMaemyyaeMaemyEaKNaemyzauMaem4Camhabeaakiabg2da9iabigdaXiabgkHiTmaaqahabaWaa8qeaeaacqWGqbaudaqadaqaaiabdogaJnaaBaaaleaacqWGPbqAaeqaaaGccaGLOaGaayzkaaGaemiCaa3aaeWaaeaacqWG4baEcqGG8baFcqWGJbWydaWgaaWcbaGaemyAaKgabeaaaOGaayjkaiaawMcaaiabdsgaKjabdIha4bWcbaGaem4qam0aaSbaaWqaaiabdMgaPbqabaaaleqaniabgUIiYdaaleaacqWGPbqAcqGH9aqpcqaIXaqmaeaacqWGmbata0GaeyyeIuoaaaa@5434@

where *C*_*i *_is the region where class *i *has the highest posterior. The probability of classification error of any classifier is lower bounded by the Bayes error [63,64]. However, the computation of the Bayes error is quite complicated. This is due to the fact that the Bayes error is obtained by integrating high-dimensional density functions in complex regions. Therefore, attention has focused on approximations and bounds for the Bayes error. One of these bound estimations for the Bayes error is provided by the Bhattacharyya distance.

In this study, we will use the Bhattacharyya distance to control the Bayes error for gene selection. For simplicity, we consider a binary classification study with *m *sample subjects and *n *measured genes. Assume there are two classes. Let *x*_*ij *_be the expression measurement of the *j*th gene for the *i*th sample, where *j *= 1, 2,..., *n*, *i *= 1, 2,..., *m*. Here we assume ***x***_**1**_,..., ***x***_***m ***_are the *m *samples, where ***x***_***i ***_= [*x*_*i*1_, *x*_*i*2_,..., *x*_*in*_]. Let ***Y ***= [*y*_1_,..., *y*_*m*_]^T ^denote the class labels of *m *samples, where *y*_*i *_= *k *indicates the sample *i *belonging to class *k *(*k *= 1, 2 stands for two different kinds of phenotypes, e.g., disease and control).

X=[Gene1Gene2⋯Genenx11x21x12x22⋯x1nx2n⋮⋮⋱⋮xm1xm2⋯xmn] and Y=[Labely1y2⋮ym]
 MathType@MTEF@5@5@+=feaafiart1ev1aaatCvAUfKttLearuWrP9MDH5MBPbIqV92AaeXatLxBI9gBaebbnrfifHhDYfgasaacH8akY=wiFfYdH8Gipec8Eeeu0xXdbba9frFj0=OqFfea0dXdd9vqai=hGuQ8kuc9pgc9s8qqaq=dirpe0xb9q8qiLsFr0=vr0=vr0dc8meaabaqaciaacaGaaeqabaqabeGadaaakeaacqWGybawcqGH9aqpdaWadaqaauaabeqaeqaaaaaabaGaem4raCKaemyzauMaemOBa4MaemyzauMaeGymaedabaGaem4raCKaemyzauMaemOBa4MaemyzauMaeGOmaidabaGaeS47IWeabaGaem4raCKaemyzauMaemOBa4MaemyzaugcbeGae8NBa4gaeaqabeaacqWG4baEdaWgaaWcbaGaeGymaeJaeGymaedabeaaaOqaaiabdIha4naaBaaaleaacqaIYaGmcqaIXaqmaeqaaaaakqaabeqaaiabdIha4naaBaaaleaacqaIXaqmcqaIYaGmaeqaaaGcbaGaemiEaG3aaSbaaSqaaiabikdaYiabikdaYaqabaaaaOqaaiabl+UimbabaeqabaGaemiEaG3aaSbaaSqaaiabigdaXiabd6gaUbqabaaakeaacqWG4baEdaWgaaWcbaGaeGOmaiJaemOBa4gabeaaaaGcbaGaeSO7I0eabaGaeSO7I0eabaGaeSy8I8eabaGaeSO7I0eabaGaemiEaG3aaSbaaSqaaiabd2gaTjabigdaXaqabaaakeaacqWG4baEdaWgaaWcbaGaemyBa0MaeGOmaidabeaaaOqaaiabl+UimbqaaiabdIha4naaBaaaleaacqWGTbqBcqWGUbGBaeqaaaaaaOGaay5waiaaw2faaiabbccaGiabbggaHjabb6gaUjabbsgaKjabbccaGiabdMfazjabg2da9maadmaabaqbaeqabuqaaaaabaGaemitaWKaemyyaeMaemOyaiMaemyzauMaemiBaWgabaGaemyEaK3aaSbaaSqaaiabigdaXaqabaaakeaacqWG5bqEdaWgaaWcbaGaeGOmaidabeaaaOqaaiabl6UinbqaaiabdMha5naaBaaaleaacqWGTbqBaeqaaaaaaOGaay5waiaaw2faaaaa@8E99@

In general, gene selection is to select relevant genes and remove redundant genes. Hence our method is divided into two steps. The first step, *Gene preselection*, is to select relevant candidate genes. The second step, *Redundancy filter*, is to apply the criterion of controlling the upper bound of the Bayes error to the remaining genes obtained from the first step for eliminating the redundant genes.

### Gene preselection

An intrinsic problem with microarray data is that sample size *m *is much smaller than the dimensionality of the genes. Our gene preselection is based on its strength for phenotype discrimination of each individual gene *j*, with *j *∈ {1, 2,..., *n*}. We use a univariate criterion function (e.g. Wilcoxon test or F-test) to evaluate discriminative power for each gene:

*Score*(*j*) = *S*(*j*)   *j *∈ {1,..., *n*}

where *S(·) *is the criterion function. We then select those relevant candidate genes according to FWER.

### Redundancy filter

We use Bhattacharyya distance to estimate the upper bound of the Bayes error, which will be used as a criterion to filter out redundant genes from remaining genes derived from *Gene preselection step*. Before discussing this step, let us introduce the Bhattacharyya distance and the relationship between the Bhattacharyya distance and the Bayes error. The Bhattacharyya distance, *d*_*B*_, can be a separability measure between two classes and also can give lower and upper bounds of the Bayes error [36]. The Bhattacharyya distance is given as:

dB=18(M2−M1)T[∑1+∑22]−1(M2−M1)+12ln⁡|(∑1+∑2)/2||∑1|1/2|∑2|1/2
 MathType@MTEF@5@5@+=feaafiart1ev1aaatCvAUfKttLearuWrP9MDH5MBPbIqV92AaeXatLxBI9gBaebbnrfifHhDYfgasaacH8akY=wiFfYdH8Gipec8Eeeu0xXdbba9frFj0=OqFfea0dXdd9vqai=hGuQ8kuc9pgc9s8qqaq=dirpe0xb9q8qiLsFr0=vr0=vr0dc8meaabaqaciaacaGaaeqabaqabeGadaaakeaacqWGKbazdaWgaaWcbaGaemOqaieabeaakiabg2da9maalaaabaGaeGymaedabaGaeGioaGdaaiabcIcaOiabd2eannaaBaaaleaacqaIYaGmaeqaaOGaeyOeI0Iaemyta00aaSbaaSqaaiabigdaXaqabaGccqGGPaqkdaahaaWcbeqaaiabdsfaubaakmaadmaabaWaaSaaaeaadaaeqaqaaiabgUcaRmaaqababaaaleaacqaIYaGmaeqaniabggHiLdaaleaacqaIXaqmaeqaniabggHiLdaakeaacqaIYaGmaaaacaGLBbGaayzxaaWaaWbaaSqabeaacqGHsislcqaIXaqmaaGccqGGOaakcqWGnbqtdaWgaaWcbaGaeGOmaidabeaakiabgkHiTiabd2eannaaBaaaleaacqaIXaqmaeqaaOGaeiykaKIaey4kaSYaaSaaaeaacqaIXaqmaeaacqaIYaGmaaGagiiBaWMaeiOBa4wbaeaabiqaeaqaamaaemaabaWaaSGbaeaadaqadaqaamaaqababaGaey4kaScaleaacqaIXaqmaeqaniabggHiLdGcdaaeqaqaaaWcbaGaeGOmaidabeqdcqGHris5aaGccaGLOaGaayzkaaaabaGaeGOmaidaaaGaay5bSlaawIa7aaqaamaaemaabaWaaabeaeaaaSqaaiabigdaXaqab0GaeyyeIuoaaOGaay5bSlaawIa7amaaCaaaleqabaGaeGymaeJaei4la8IaeGOmaidaaOWaaqWaaeaadaaeqaqaaaWcbaGaeGOmaidabeqdcqGHris5aaGccaGLhWUaayjcSdWaaWbaaSqabeaacqaIXaqmcqGGVaWlcqaIYaGmaaaaaaaa@719D@

***M***_***k ***_is the mean vector of class *k *(*k *= 1 or 2); ∑_***k ***_is the covariance matrix of class *k *(*k *= 1 or 2). The first term of Equation (2) gives the class separability due to the difference between class means, and the second term gives the class separability due to the difference between class covariance matrices. The Bayes error of classification between the two classes is bounded by the following expression:

εB≤P1P2exp⁡(−dB)
 MathType@MTEF@5@5@+=feaafiart1ev1aaatCvAUfKttLearuWrP9MDH5MBPbIqV92AaeXatLxBI9gBaebbnrfifHhDYfgasaacH8akY=wiFfYdH8Gipec8Eeeu0xXdbba9frFj0=OqFfea0dXdd9vqai=hGuQ8kuc9pgc9s8qqaq=dirpe0xb9q8qiLsFr0=vr0=vr0dc8meaabaqaciaacaGaaeqabaqabeGadaaakeaaiiGacqWF1oqzdaWgaaWcbaGaemOqaieabeaakiabgsMiJoaakaaabaGaemiuaa1aaSbaaSqaaiabigdaXaqabaGccqWGqbaudaWgaaWcbaGaeGOmaidabeaaaeqaaOGagiyzauMaeiiEaGNaeiiCaa3aaeWaaeaacqGHsislcqWGKbazdaWgaaWcbaGaemOqaieabeaaaOGaayjkaiaawMcaaaaa@3F40@

where *P*_*k *_is prior probability of class *k *(*k *= 1 or 2). We can derive the upper bound of the Bayes error evaluated from the Inequality (3) with *P*_1 _= *P*_2 _= 0.5. That is,

εB∗
 MathType@MTEF@5@5@+=feaafiart1ev1aaatCvAUfKttLearuWrP9MDH5MBPbIqV92AaeXatLxBI9gBaebbnrfifHhDYfgasaacH8akY=wiFfYdH8Gipec8Eeeu0xXdbba9frFj0=OqFfea0dXdd9vqai=hGuQ8kuc9pgc9s8qqaq=dirpe0xb9q8qiLsFr0=vr0=vr0dc8meaabaqaciaacaGaaeqabaqabeGadaaakeaaiiGacqWF1oqzdaqhaaWcbaGaemOqaieabaGaey4fIOcaaaaa@3083@ = 0.5 exp(-*d*_*B*_)

We will use ε*_B _to control the Bayes error in classification analyses in order to filters out redundant genes. After the gene preselection procedure, the remaining genes form the candidate gene subset, B, which is regarded as an informative gene set. Then we construct an empty set, A, for selected relevant genes. We select the gene ranked first in the list of B as the initial gene in A. The gene ranked first is much more informative than any other genes to discriminate the two classes, thus we set it as the indispensable gene in A. We then use Sequential Forward Selection algorithm to select the genes with great contribution to the Bhattacharyya distance between two classes [34]. When the estimated upper bound of Bayes error reach pre-defined criterion, the search procedure stops and return the selected gene set, A.

In summary, our algorithm for gene selection proceeds as follows:

Step 1: Use a criterion function (we adopt Wilcoxon test in this study) to evaluate the discriminative power for each gene and select candidate genes according to FWER level.

Step 2: 1) Initialize A as an empty set (A is the set of selected relevant genes)

2) Initialize B as the candidate genes set, pick one gene ranked first in the list of criterion function values from B and put it into A as an initial gene.

3) For *i *= 2: *t *(*t *is the number of genes to be selected)

• for *j *= 1:*q *(*q *is the number of genes in B)

---Take gene *j *from B, put it into A, and calculate *d*_*B*_(*j*) with all genes in A.

• end

• Select the gene from B with the maximal *d*_*B *_value and calculate corresponding ε*_B_

• if ε*_B _is greater than or equal to pre-defined criterion (here this criterion is set as 1.0E-4, which will be discussed later in Discussion section)

----Put this gene into set A; remove this gene from B

• else

----Stop the cycle and return the gene set A

4) End

Implementation of BBF method is available per request from zhangjig@umkc.edu with source code in R.

## Competing interests

The author(s) declares that there are no competing interests.

## Authors' contributions

JGZ developed the algorithm and performed the analyses for five real-data sets. HWD provided discussion, general supervision and revised the manuscript for this study. Both authors have approved the final version of the manuscript.
